# Study on X-ray Emission Using Ultrashort Pulsed Lasers in Materials Processing

**DOI:** 10.3390/ma14164537

**Published:** 2021-08-12

**Authors:** Joerg Schille, Sebastian Kraft, Theo Pflug, Christian Scholz, Maurice Clair, Alexander Horn, Udo Loeschner

**Affiliations:** 1 Laserinstitut Hochschule Mittweida, University of Applied Sciences Mittweida, Technikumplatz 17, 09648 Mittweida, Germany; kraft@hs-mittweida.de (S.K.); pflug@hs-mittweida.de (T.P.); horn4@hs-mittweida.de (A.H.); loeschne@hs-mittweida.de (U.L.); 2 3D-Micromac AG, Technologie-Campus 8, 09126 Chemnitz, Germany; scholz@3d-micromac.com (C.S.); clair@3d-micromac.com (M.C.)

**Keywords:** laser, ultrashort pulse, plasma, X-ray, dose rate, Bremsstrahlung, resonance absorption, burst, bi-burst

## Abstract

The interaction of ultrashort pulsed laser radiation with intensities of 10^13^ W cm^−2^ and above with materials often results in an unexpected high X-ray photon flux. It has been shown so far, on the one hand, that X-ray photon emissions increase proportionally with higher laser power and the accumulated X-ray dose rates can cause serious health risks for the laser operators. On the other hand, there is clear evidence that little variations of the operational conditions can considerably affect the spectral X-ray photon flux and X-ray emissions dose. In order to enhance the knowledge in this field, four ultrashort pulse laser systems for providing different complementary beam characteristics were employed in this study on laser-induced X-ray emissions, including peak intensities between 8 × 10^12^ W∙cm^−2^ < *I*_0_ < 5.2 × 10^16^ W∙cm^−2^, up to 72.2 W average laser power as well as burst/bi-burst processing mode. By the example of AISI 304 stainless steel, it was verified that X-ray emission dose rates as high as ${\dot{H}}^{\prime }\;\left(0.07\right)$ > 45 mSv h^−1^ can be produced when low-intensity ultrashort pulses irradiate at a small 1 µm intra-line pulse distance during laser beam scanning and megahertz pulse repetition frequencies. For burst and bi-burst pulses, the second intra-burst pulse was found to significantly enhance the X-ray emission potentially induced by laser pulse and plasma interaction.

## 1. Introduction

Ultrashort pulse lasers feature excellent performances for high-efficient and precision machining that also has attracted increasing attention for innovations in micro fabrication and surface engineering. Moreover, the recent power scaling of femtosecond (fs) and picosecond (ps) lasers reaching multi-kW class level [1,2] will pave the way of the ultrashort pulse laser technology from the laboratory into industrial production. Actually, in typical material processing scenarios, ultrashort laser pulses of micro to milli joules optical energy and hundreds of femtoseconds to picoseconds pulse durations are employed, for example, in laser micro drilling [3,4], laser engraving [5,6], or laser surface texturing and modifications [7,8]. By tightly focusing the laser radiation, high peak intensities in the range from 10^13^ to 10^16^ W∙cm^−2^ can be reached on the material surface. However, when using laser pulses of such high intensity in material ablation, high-intense electron plasmas will be produced, itself emitting X-ray photons at a several keV level. The X-ray photons result from Bremsstrahlung and characteristic line emissions when accelerated electrons lose their kinetic energy by scattering, acceleration and recombination with other charged particles, in free–free, free–bound, and bound–bound processes.

However, recent works have shown that the X-ray photon emission increases proportionally with higher average laser power, and actual X-ray emission dose rates can cause serious health risks for the laser operators [9,10,11,12,13,14]. In particular, this is the case when high-pulse repetition frequency lasers will be used in materials ablation as the X-ray emission per pulse can accumulate to harmful X-ray photon dose over time. On the one hand, it is verified that X-ray photon emission increases with peak intensity, pulse repetition rate, and average laser power. On the other hand, there is clear evidence that little variations of the operational conditions can have a considerable effect on the X-ray emission, causing unexpected high dose levels. This is much more relevant, as a great number of parameters (more than 20) have been experimentally validated as influencing factors on X-ray emission dose level, including laser beam characteristics, processing conditions, and the irradiated material, further mutually affecting each other. Moreover, the specific effect of each parameter and their variations is not sufficiently investigated and understood so far, making it difficult for the reliable assessment of the X-ray emission and safety precautions in the operational area.

In the following, the significant impact of the pulse repetition frequency and the intra-line pulse distance during laser beam scanning on the spectral X-ray photon flux and the X-ray emission dose is discussed by comparing low-intensity and high-intensity ultrashort pulses irradiating AISI 304 stainless steel. It will be shown that an unexpected high level of X-ray emission dose rate of tens of mSv∙h^−1^ could be produced when low-intensity pulses irradiate at 1.6 MHz pulse repetition frequency (PRF). This resulted potentially from plasma resonance absorption as an efficient mechanism for optical energy transfer, also yielding high X-ray emissions in the burst- and bi-burst pulse regime.

## 2. Materials and Methodology

**Figure 1:**
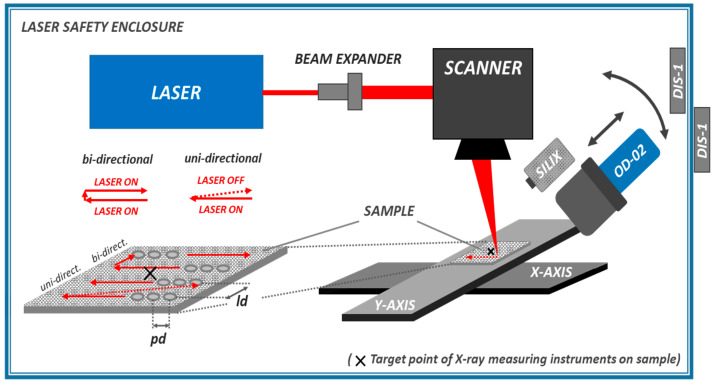
Schematic view of the laser safety enclosure including laser machining setup.

All experiments were performed in a laser safety enclosure to protect the experimental operators from potential risks and hazards arising from the powerful laser beams and X-ray emissions. A schematic view of the laser machining setup used in this study is presented in Figure 1.

First of all, the efficacy of the enclosure material was verified by initial testing in order to ensure reasonable assurance of the enclosure against the laser-induced X-ray emissions. In addition, three electronic Direct Ion Storage memory cells (DIS-1, Mirion Technologies (Canberra) GmbH, Ruesselsheim, Germany) were applied as safety precautions. Two of the X-ray badge detectors were placed at the inner and outer sides of the laser safety enclosure. A third one was worn by the operator throughout the experiments. The actual received X-ray dose values were read out at the beginning and the end of each working day from the badges. It should be mentioned here that no increase of the X-ray dose could be detected, whether from the badges placed at the enclosure outside nor the one worn by the operator, confirming the safe working conditions at all time. 

In total, four different laser systems were employed in this study for providing a wide range of complementary laser beam characteristics; see Table 1. The studied parameter settings include up to 5.2 × 10^16^ W∙cm^−2^ peak intensity, pulse repetition frequencies in the range between 1 and 2 MHz, maximum 72.2 W average laser power, as well as laser burst and bi-burst processing mode. The energy of the irradiating pulses *Q*_P_ was defined by the ratio of the average laser power *P*_av_ and the pulse repetition frequency *f*_P_ described by:

(1) $$Q_{P}=\frac{P_{\mathrm{av}}}{f_{P}}.$$

The focus spot diameters *d*_86_ (86.5% power enclosure method) presented in Table 1 have been determined by analyzing the laser beam caustic (MSM Micro Spot Monitor, Primes GmbH, Pfungstadt, Germany) or rather from the established semi-log plot method of squared ablation crater diameter versus peak fluence [15].

The X-ray emission induced by the ultrashort pulsed laser irradiations was studied on technical-grade AISI 304 stainless steel metal sheets. The chemical composition of the alloying elements is presented in Table 2. The dimensions of the laser processed substrates were 50 × 50 × 2 mm³ in width, length, and sample thickness. In each test, the substrates were placed on a sample holder in the focal plane with the help of an XYZ stage assembly. Two different machining strategies were applied depending on the prevailing machinery conditions and setups of the individual laser systems: (i) relative movements between the laser beam and substrate by accelerating the sample in XY directions; and (ii) scanning the laser radiation across the substrate surface via a galvanometer scan system (intelliScan 30, Scanlab GmbH, Puchheim, Germany). As illustrated in Figure 1, the geometrical distance of the irradiating pulses along and between the laser processed lines is defined by the intra-line pulse distance *pd* (as a function of laser beam moving speed and pulse repetition frequency) and the hatch distance *ld*, respectively. The laser pulse peak power *P*_0_ provided in Table 1 for the investigated laser beams was calculated according to

(2) $$P_{0}=\frac{Q_{P}}{c\;\cdot \;\tau_{H}}$$

where the pulse energy *Q*_P_ is divided by the measured pulse duration *τ*_H_ and by taking into account the correction factor *c* considering the temporal pulse shape of the respective irradiating pulses, either being Gaussian or sech^2^. Following

(3) $$I_{0}=\frac{2\cdot P_{0}}{\pi \;\cdot \;w_{0.86}{}^{2}},$$

the peak intensity *I*_0_ was calculated from the peak power and the focus spot radius *w*_0.86_.

Two different detector systems were used for the X-ray photon emission measurements: a survey meter OD-02 (STEP Sensortechnik und Elektronik Pockau GmbH, Pockau-Lengefeld, Germany) measuring the directional X-ray emission dose *H’*(0.07) and the X-ray emission dose rate equivalent ${\dot{H}}^{\prime }$(0.07) = d*H’*(0.07)/dt in the soft-radiation region above 6 keV. Additionally, a SILIX lambda detector (Ingenieurbüro Prof. Dr.-Ing. Guenter Dittmar, Aalen, Germany) monitored the X-ray emission spectra and X-ray emission dose rate in the photon energy range between 2 and 20 keV. The X-ray detectors were aligned to the center of the samples, which is indicated in Figure 1 by the target point (X). The detector’s view was along the *X*-axis equivalent to the laser processing direction. The nominal detector distance *D* and the detection angle α were varied with reference to the target point and the horizontal plane. Thereby, the nominal detector distance represented a mean measure because the detector target points were set in the center of the laser processed area. Hence, in the detector’s view direction (*X*-axis), the closest point and most distant point from the sensitive detector surface were at the start and end of a laser scanned line, and likewise the greatest distance in the *Y*-direction was at the edges of the laser processed fields. 

Laser beam scanning was applied either in uni-directional or bi-directional mode depending on the specifics of the studied laser setups in terms of laser beam control and synchronization. In uni-directional processing, the laser beam departs from the X-ray detector, thus receiving maximum X-ray dose levels [13]. The “laser-on” duty cycle was 46% in uni-directional processing controlled by oscilloscope measurements throughout the study. As a consequence, the monitored X-ray emissions represented less than half of the process-relevant emission dose and were corrected (by a factor of 2.17) in order to compare and discuss the process-typical dose rates in the following. In bi-directional mode, by contrast, the laser beam was switched on during all the processing. Hence, the monitored X-ray emissions represent average readings from the bi-directional laser material processing. This is due to the fact that higher X-ray emissions have been reported for a laser beam running away instead of moving toward the sensitive detector surface [13]. 

## 3. Results and Discussions

### 3.1. Effect of the Detection Angle and Detector Distance on X-ray Dose

At first, the dependencies of detector’s angle and distance on the monitored X-ray emission dose rate were studied. This was in order to identify the optimum measuring conditions for the assessment of X-ray emissions in the accomplished study. On the one hand, this was encouraged, as the authors´ literature review revealed a dependence between the X-ray emission dose and the detection angle [9]. So far, a maximum X-ray dose rate level was reported at a 30° detection angle, as investigated in the range between 10° and 40°. On the other hand, the correct detector–substrate distance is of relevance because, firstly, the X-ray photons are absorbed in air, and secondly, to ensure that the active detector area is homogeneously exposed by the laser-induced X-ray emissions. Both have a great impact on the detector readings. 

**Figure 2:**
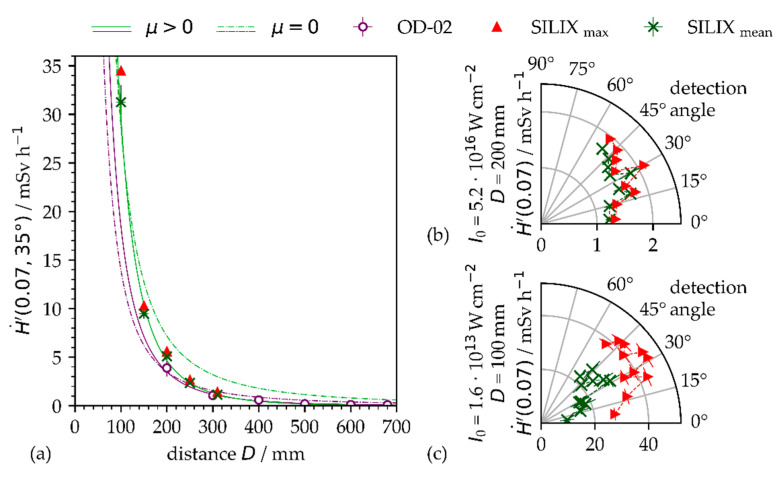
(**a**) OD-02 and SILIX readings of X-ray emission dose rate on AISI 304 as a function of detector distance *D*; the fitting curves are representative for vacuum (dashed line) and air (solid line) ambient conditions at *I*_0_ = 1.6 × 10^13^ W∙cm^−2^ and 35° detection angle. (**b**) X-ray emission dose rate as a function of detection angle for pulses of *I*_0_ = 5.2 × 10^16^ W∙cm^−2^ at 200 mm SILIX detector distance. (**c**) X-ray emission dose rate as a function of detector angle for pulses of *I*_0_ = 1.6 × 10^13^ W∙cm^−2^ at 100 mm SILIX detector distance.

The X-ray emissions recorded by the survey meter OD-02 and the SILIX X-ray monitor at a 35° detector angle and different distances from the substrate can be seen in Figure 2a at similar dose values. A fitting curve (dashed line) is included in the plot, showing the recorded X-ray emission dose rate inversely proportional to the square of the distance *D* of the detector from the substrate target point. However, at the larger distances, the experimental data differ to a greater extent from the inverse square-root relationship resulting from X-ray beam absorption in air. This is pointed out in Figure 2a by the modified fitting curve (solid line) emphasizing good agreement for experimental and computed data when the attenuation coefficient *µ* is considered in the photon flux Φ_phot_, calculations

(4) $$\Phi_{\mathrm{phot}}\left(D,\lambda \right)\propto D^{-2}e^{-\mu \left(\lambda \right)D}.$$

Thereby, the attenuation coefficient depends strongly on the photon energy and varies in air between 0.94 cm^−1^ ≤ µ ≤ 6.36 × 10^2^ cm^−1^ for the X-ray photons between 2 keV ≤ E_phot_ ≤ 20 keV [10]. The attenuation coefficients derived from the fitting curves presented in Figure 2a amount to µ = 0.05 ± 0.02 cm^−^^1^ (SILIX) or µ = 0.03 ± 0.02 cm^−^^1^ (OD-02), which is about two orders of magnitude lower than the literature data. However, this discrepancy is not clarified yet; the steadily changing distance of the actual laser processing zone to the detector resulting from laser beam scanning, the X-ray dose rate as an integral measure from the X-ray photon spectrum, and measurement uncertainties for X-ray emissions all had a great impact on the experimental data.

In addition, a significant effect of the detector angle on the X-ray emission dose rate has been found by varying the angular orientation of the SILIX detector in the range between 7.0° < α < 51.0° with respect to the specified target point. Most likely independent from the peak intensity, whether *I*_0_ = 5.2 × 10^16^ W∙cm^−2^ (Figure 2b) or *I*_0_ = 1.6 × 10^13^ W∙cm^−2^ (Figure 2c), the maximum X-ray emission dose rate was detected at about 30°. It is noteworthy that a considerably higher X-ray emission dose rate was measured for the pulses of lower intensity, 43 mSv∙h^−1^ vs. 2.1 mSv∙h^−1^, potentially resulting from the higher average laser power, 72.2 W vs. 1.6 W, and shorter distance of the detector from the target point, 100 mm vs. 200 mm.

### 3.2. Spectral X-ray Emission

**Figure 3:**
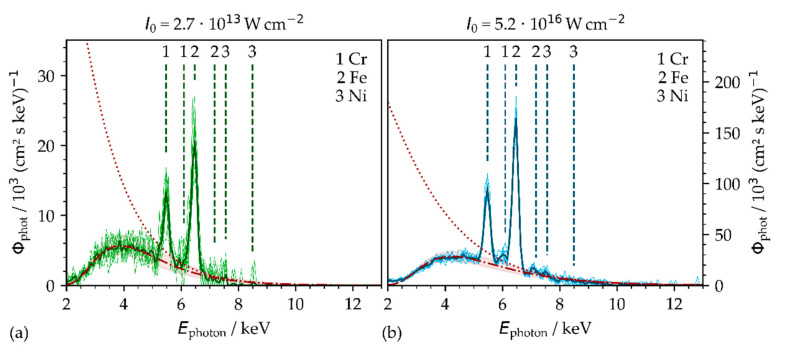
X-ray photon flux $\Phi_{\mathrm{phot}}\;$ at a 35° detection angle and 100 mm distance for low peak intensity (**a**) *I*_0_ = 2.7 × 10^13^ W·cm^−2^ and high peak intensity (**b**) *I*_0_ = 5.2 × 10^16^ W·cm^−2^. The characteristic X-ray emission lines of the correlating AISI 304 alloying elements are included. The recorded spectral X-ray photon distribution follows a Maxwell–Boltzmann distribution computed for vacuum (dotted line, non-attenuated) and ambient air (dashed line including 3 σ confidence interval, 100 mm distance) conditions.

The spectral X-ray photon flux was analyzed by placing the SILIX X-ray monitor at a fixed 35° detection angle and 100 mm distance from the target point at the substrates. Two representative X-ray photon emission spectra in Figure 3 verify a broad continuous Bremsstrahlung emission in the photon energy range below 10 keV. The detected characteristic line emissions can be related to the main AISI 304 alloying elements. Therefore, as a reference, the principal K_α__1-3_ and K_β__1-5_ shell interband emissions are included in Figure 3, such as for iron (Fe), chromium (Cr), and nickel (Ni), as provided by the NIST database [16]. By comparing the individual characteristic lines, the photon energy peaks of the alloying elements are slightly offset from the principal X-ray transition energies (Δ*E*_ph_ < 0.09 keV) to higher photon energy levels being within the measurement accuracy of the SILIX X-ray monitor.

The monitored Bremsstrahlung spectra represent qualitatively the energy distribution of the X-ray photon flux at 100 mm distance and can be approximated by a Maxwell–Boltzmann distribution [9,12] according to

(5) $$f_{Maxwell}\left(E\right)dE=\sqrt{\frac{4E}{\pi {\left(k_{B}T_{e}\right)}^{3}}}\;e^{-\frac{E}{k_{B}T_{e}}}\;dE$$

where *k*_B_ represents the Boltzmann constant, *T*_e_ is the electron temperature, and *E* is the energy of the emitted X-ray photons.

The recorded Bremsstrahlung continuum can be seen in good correlation with the computed X-ray emissions when considering the transmission of the X-rays through air at ambient pressure and 100 mm distance in the Maxwell–Boltzmann modeling procedure (Figure 3, dashed line). In addition, the non-attenuated spectrum computed for vacuum surrounding conditions is presented (dotted line). The best fit between the empirically observed and theoretically modeled X-ray photon distribution (in air) was obtained by the electron temperature of either 1.18 ± 0.1 keV (Figure 3a) for the low-intensity or 1.56 ± 0.1 keV (Figure 3b) for the high-intensity pulses. These electron temperatures are in good agreement with the temperatures reported elsewhere for hot electrons and respective laser peak intensities ranging between 0.82 and 1.76 keV [9,15,17].

The averaged X-ray emission dose rate of the above given spectra was measured to ${\dot{H}}^{\prime }\left(0.07\right)$ = 1.1 ± 0.2 mSv·h^−1^ with the low peak intensity pulses (*I*_0_ = 2.7 × 10^13^ W·cm^−2^), as shown in Figure 3a, and ${\dot{H}}^{\prime }\left(0.07\right)$ = 11.4 ± 2.3 mSv·h^−1^ with the high peak intensity pulses (*I_0_* = 5.2 × 10^16^ W·cm^−2^); see Figure 3b. However, for the high peak intensity pulses, the X-ray emission rate was about one order of magnitude higher in spite of the considerably lower applied average laser power (22.4 W vs. 1.6 W) and PRFs (506 kHz vs. 1 kHz). This finding proves the great impact of the laser peak power and intensity on the X-ray emission and will be discussed more in detail in the following section.

### 3.3. X-ray Emission in the Low-Intensity and High-Intensity Pulse Regime

The effect of the peak intensity on the X-ray emission was investigated by recording the X-ray photon spectra at 100 mm distance and 35° detection angle of the SILIX monitor from the reference target point at the substrates. The peak intensity of the impinging laser pulses was varied in the range between 8 × 10^12^ W·cm^−2^ < *I*_0_ < 1.6 × 10^13^ W·cm^−2^ and 0.3 × 10^16^ W·cm^−2^ < *I*_0_ < 5.2 × 10^16^ W·cm^−2^ that also refers to the low-intensity and high-intensity pulse regime in the following.

**Figure 4:**
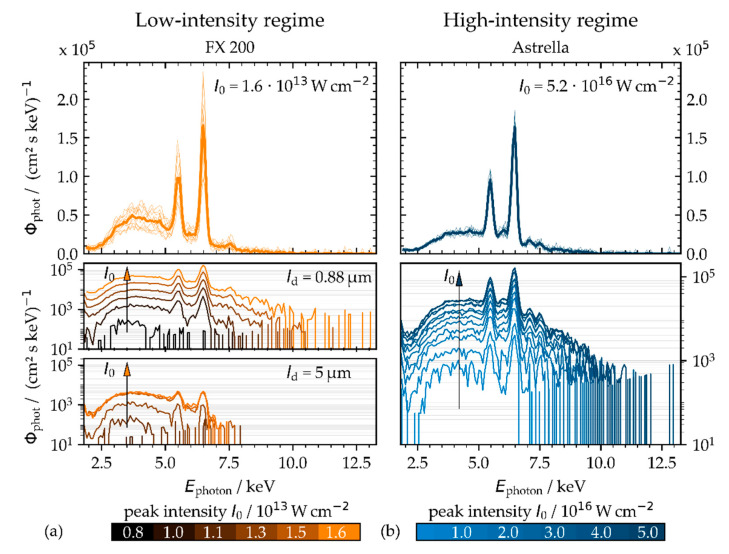
X-ray photon flux $\Phi_{\mathrm{phot}}\;$ detected on AISI 304 in the low-intensity (**a**) and high-intensity pulse regime (**b**). The X-ray emission spectra were recorded by using the SILIX X-ray monitor at 35° detection angle and 100 mm distance. In the low-intensity regime, the intra-line pulse distance was varied from 0.88 µm (left, center) to 5 µm (left, bottom). Increasing peak intensity is indicated by the arrow.

A closer view to the X-ray emission spectra in Figure 4 reveals a notably higher extent of Bremsstrahlung emissions for pulses of lower peak intensity, while the greater proportional amount of characteristic X-ray emissions can be seen for pulses of higher intensity. The corresponding X-ray emission dose rates were measured of ${\dot{H}}^{\prime }\left(0.07\right)$ = 38.9 ± 4.6 mSv·h^−1^ for pulses of 1.6 × 10^13^ W·cm^−2^ and ${\dot{H}}^{\prime }\left(0.07\right)$ = 11.4 ± 2.3 mSv·h^−1^ for 5.2 × 10^16^ W·cm^−2^ peak intensity. This was achieved by irradiating a laser beam of 72.2 W and 1.6 MHz (low-intensity regime) or rather 1.6 W and 1 kHz average laser power and pulse repetition frequency (high-intensity regime), respectively.

The differences of the X-ray photon spectra can be attributed to the different mechanisms of laser plasma interaction and electron plasma heating that largely depend on the irradiating conditions. So, pulses of lower intensity (*I*_0_ < 10^14^ W·cm^−2^) will be primarily absorbed in the underdense corona region of the plasma by inverse Bremsstrahlung absorption processes [18]. The excited electrons will be accelerated in the Coulomb field that resulted in the emission of the Bremsstrahlung continuum. This free–free generation of Bremsstrahlung X-ray photons is inefficent and yields only a low proportion of X-ray photons per laser pulse. Certainly, a great amount of low-intensity pulses can induce X-rays at a high dose level, as can be seen in Figure 4a (top) by the example of low-intensity pulses (*I*_0_ = 1.6 × 10^13^ W·cm^−2^) irradiated at 1.6 MHz pulse repetition frequency.

By contrast, resonance absorption is suggested as the dominant mechanism for pulses of high peak intensity, *I*_0_ > 10^15^ W·cm^−2^. This collision-less process is most efficient for parallel polarized laser beams irradiating at a large incident angle. The laser energy will be transferred to the electron plasma in the region of the critical plasma density, where the electron plasma frequency is equal to the laser frequency [19]. The highly excited plasma electrons interact with the bound electrons of the target atoms by collisional impact ionization, leaving vacant energy levels. Nevertheless, resonance absorption might also be effective when pulses of lower peak intensity irradiate at megahertz PRF and small spatial pulse distance. This can be supposed from the X-ray emission spectra presented in Figure 4a (center and bottom) recorded for pulses of the same intensity (1.6 × 10^13^ W·cm^−2^) and PRF (1.6 MHz) but different intra-line pulse distances varied by the laser beam moving speed. As characteristic X-ray emission lines are clearly distinguishable in the spectra recorded at an 0.88 µm intra-line pulse distance, the Bremsstrahlung continuum is dominant at the larger 5 µm intra-line pulse distance.

In addition, there is a trend toward higher X-ray photon flux when pulses of higher peak intensity irradiate, as clearly observable in Figure 4 for the low-intensity (left) and the high-intensity (right) pulse regime. On the one hand, the displayed spectra indicate no significant dependence of the characteristic X-ray emissions from the iron and chromium alloy elements from the peak intensity. On the other hand, the spectral width of the Bremsstrahlung continuum enlarged to higher X-ray photon energy with pulses of higher peak intensity, such as 10.0 keV or rather 12.0 keV in maximum for the low-intensity (*I*_0_ = 1.6 × 10^13^ W·cm^−2^) or high-intensity (*I*_0_ = 5.2 × 10^16^ W·cm^−2^) pulse regime. Here, too, the effect of the intra-line pulse distance on the X-ray photon emission spectrum can be recognized in Figure 4a, showing 11.0 keV maximum X-ray photon energy at 0.88 µm, while the maximum of about 7.5 keV can be seen for the 5.0 µm intra-line pulse distance.

**Figure 5:**
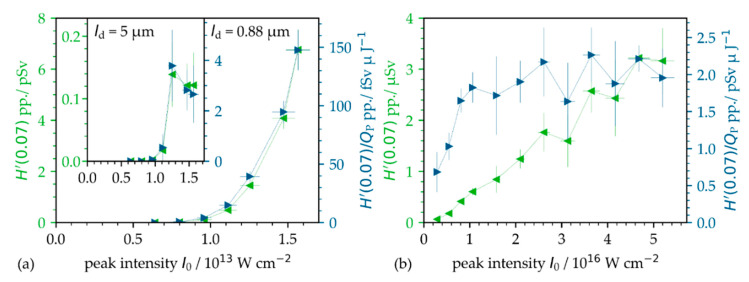
X-ray dose per pulse (green data points) and the X-ray emission efficiency (blue data points) on AISI 304 in the (**a**) low-intensity and (**b**) high-intensity regime. The given data were derived from the X-ray emission dose monitored by the SILIX detector at a 35° detection angle and 100 mm distance. The dotted lines guide the eye.

The emitted X-ray dose per pulse (Figure 5, green data points) was derived by dividing the monitored X-ray emission dose rate by the number of impinging pulses. In addition, as another evaluation measure, the X-ray emission efficiency represents the X-ray dose per pulse in relation to the applied pulse energy; see Figure 5 (blue data points). In the low-intensity pulse regime, the X-ray dose per pulse increases exponentially with higher peak intensity, and in the studied range, a maximal value of 6.7 pSv/pulse was measured at *I*_0_ = 1.6 × 10^13^ W·cm^−2^ and *f*_P_ = 1.6 MHz; see Figure 5a. This is in contrast to the high-intensity regime, as shown in Figure 5b, where the X-ray dose per pulse increases linearly with increasing peak intensity. Here, the determined maximum value of the X-ray dose per pulse of 3.2 nSv/pulse is almost three orders of magnitude larger than for low-intensity pulses. In terms of X-ray emission efficiency, Figure 5a indicates an exponential dependence for the low-intensity regime to 150 fSv·µJ^−1^ maximum, while saturation is given in Figure 5b of about 2.2 pSv·µJ^−1^.

For pulses of low peak intensity irradiated at megahertz PRF, a remarkable dependence of the X-ray emission efficiency on the spatial pulse distance could be identified. The X-ray dose per pulse is at a considerably lower level with a 5.0 µm intra-line pulse distance under otherwise similar processing conditions; see Figure 5a (imprint). This might be due to the fact that the interaction of the following pulse(s) with the still prevailing laser ablation particle/plasma plume changed dramatically depending on the lateral pulse distance. The underlying physical effects are not clear yet; however, plasma resonance absorption was introduced above as a potential mechanism for the transfer of optical energy to the plasma even when pulses of low peak intensity irradiate at high PRF. This is supported by the fact that the angle of incidence between the incoming laser beam and the critical plasma density layer is continually changing with time. Hence, the actual plasma density profile (affected by the time delay between the pulses determined by the pulse repetition frequency) and the lateral offset of the next impinging pulse determine the optimum conditions for laser beam coupling and energy transfer to the plasma with a consequence of maximum X-ray photon emission. This hypothesis will be tested in the ongoing study.

For the presented data, it should be mentioned that scaling of the X-ray dose per pulse or X-ray emission efficiency with the irradiated pulse number and pulse intensity can underestimate the actual emitted X-ray dose. This is due to the fact that laser plasma and X-ray emission influencing effects are not adequately considered in the proposed assessments [12]. For example, this includes the temporal pulse distance, geometrical pulse distance, angle of incidence as well as surface inhomogeneity, and roughness effects that steadily change with ongoing laser processing and scan numbers, and all of them mutually reinforce each other. To underline this, a more detailed view on the particular effect of the geometrical and temporal distance between the irradiating pulses on X-ray emission is provided in the following sections.

### 3.4. Effect of Intra-Line Pulse Distance on X-ray Emission in the Low-Intensity Pulse Regime

**Figure 6:**
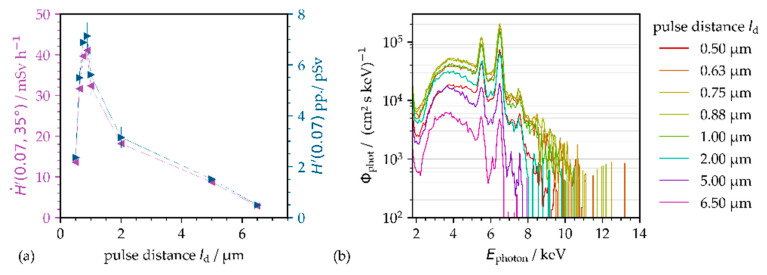
X-ray emissions on AISI 304 at 100 mm distance and 35° detection angle as induced by low-intensity pulse irradiations at *I*_0_ = 1.6 × 10^13^ W·cm^−2^, *f*_P_ = 1.6 MHz PRF, and *P*_av_ = 72.2 W average laser power. (**a**) X-ray emission dose rate and (**b**) X-ray photon spectra as a function of intra-line pulse distance. The dotted lines in (**a**) guide the eye.

A remarkable influence of the intra-line distance between the impinging ultrashort laser pulses on X-ray emission has already been reported for tungsten studied with PRFs in the range between 50 kHz < PRF < 400 kHz [10]. Pronounced X-ray emission maxima and minima have been detected where the amplitude of the X-ray doses varied by a factor up to two depending on the spatial distance of the next impinging pulse. These previous findings are supported by the results of our study as a greater effect of the intra-line pulse distance on X-ray emission was measured for AISI 304 irradiated with low-intensity pulses of *I*_0_ = 1.6 × 10^13^ W·cm^−2^ at 1.6 MHz PRF (Figure 6a). The intra-line pulse distance was varied between 0.5 µm ≤ *pd* ≤ 6.5 µm by adjusting the laser beam moving speed from 0.8 to 10.4 m·s^−1^.

The highest X-ray emission dose rate was measured of ${\dot{H}}^{\prime }\left(0.07\right)=44.9\;\mathrm{mSv}\cdot h^{-1}$ at 0.88 µm intra-line pulse distance. At smaller or larger intra-line pulse distances, the maximum X-ray emission dose reduced significantly to ${\dot{H}}^{\prime }\left(0.07\right)=18.3\;\mathrm{mSv}\cdot h^{-1}$ at 0.5 µm or rather ${\dot{H}}^{\prime }\left(0.07\right)=3.8\;\mathrm{mSv}\cdot h^{-1}$ at 6.5 µm, respectively.

For tungsten, it is reported in the literature that the X-ray emission maxima and minima resulted from multi-beam reflections at the sidewalls of the ablated grooves [10], which (influenced by the intra-line pulse distance) originated with different micro groove wall angles. Here, in our study on AISI 304, a substantial effect of the surface topography on the X-ray emission dose can largely be ruled out. This is because the pronounced X-ray emission maximum at 0.88 µm intra-line pulse distance could also be confirmed for different surface topographies (not presented here), such as those produced with the intra-line pulse distances specifically addressed in Figure 6a. A more likely explanation for the observed great X-ray emission at small intra-line pulse distance is a strong interaction of the next irradiating pulses with the still existent laser ablated plasma/nanoparticle plume. As a matter of fact, an evident laser plasma/nanoparticle ablation plume has been verified in pump-probe shadowgraph analyses for the times 0.5–1.0 µs after pulse irradiation [20]. These time intervals correlate very well to the time domain of 1.6 MHz repetitive pulses presented in Figure 6.

**Figure 7:**
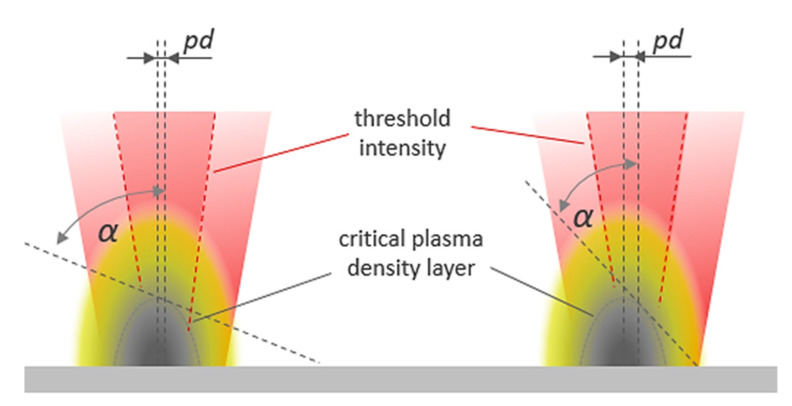
Schematic view on the effect of the intra-line pulse distance *pd* on the angle of incident of the laser beam α with respect to the flank of the critical plasma density layer.

This can be linked to the effect of the intra-line pulse distance on X-ray emission, as the geometrical spacing defines the angle of incidence for the next impinging laser pulse in relation to the flank of the apparent laser ablation plume; see Figure 7.

Nevertheless, the laser ablation plume is on a much larger dimension than the very little intra-line pulse distance of only a few micro meters, showing the greatest impact on X-ray emission in Figure 6. Therefore, on the one hand, a reasonable explanation might be that an efficient laser pulse with plasma interaction takes place close to the solid density state [21]. On the other hand, up to 80% absorption levels were reported for p-polarized laser beams under striking incidence at steep plasma density profiles by collision-less resonance absorption mechanisms [21]. Highly excited plasma states and steep plasma density gradients emerge in regions of intense laser excitation at the highest laser peak intensity being in the narrow center of the Gaussian laser beam. So, it is obvious that the effective cross-section area of the high-intensity center part of the laser beam with the critical plasma layer is considerably smaller than the interaction area of the laser beam with the induced plasma/nanoparticle plume. Hence, a variation of a few micrometers of the intra-line pulse distance changes the position of the next irradiating pulse on the flank of the critical plasma layer. In fact, it has been demonstrated so far that plasma resonance absorption is very sensitive to the specific conditions of the resonant field near the critical density surface [22]. From this, it becomes clear that the distance of the next following pulse from the critical plasma density layer could significantly affect the incident angle of the laser beam relative to the critical plasma layer flank, in turn, with a high impact on the laser plasma resonance absorption and thus optical energy transfer.

However, additional collision-less absorption processes have been identified so far for intense laser pulses interacting with overdense plasma; for example, sheath inverse Bremsstrahlung [23], non-linear mechanisms [24] or anharmonic resonance effects [25]. These absorbing processes depend strongly on the angle of incident and the polarization of the incident laser beam and cannot be ruled out to have an effect on the X-ray photon emissions detected in this study.

The higher level of X-ray emission at the smaller intra-line pulse distances is also confirmed by the X-ray emission spectra presented in Figure 6b. It can be seen that X-ray emission is dominated by the Bremsstrahlung continuum at the larger intra-line pulse distance between 2 µm < *pd* < 6.5 µm, while characteristic X-ray emission and increased X-ray photon flux is obvious for the smaller intra-line pulse distance ranging between 0.5 µm < *pd* < 1.0 µm.

The highest X-ray photon yield and most prominent characteristic line emission were measured for pulses of 0.75 µm and 0.88 µm intra-line pulse distance corresponding to the position of highest X-ray dose rate in Figure 6b. In addition, the spectral width of the monitored X-ray photon fields enlarged to higher energy with pulses of smaller intra-line pulse distance. In fact, 8.0 keV maximum photon energy was detected at a 5 µm intra-line pulse distance compared to 11.0 keV at 0.88 µm, respectively. These enlarged X-ray spectra indicate even for the low-intensity pulses irradiated at small intra-line pulse distance a higher degree of plasma ionization and, therefore, a more-efficient coupling of the irradiating low-intensity ultrashort pulses. This is supported by the fact that the enlarged X-ray photon spectra of Figure 6b could be observed similar to the ones shown above in Figure 4 for higher electron temperatures induced by high-intensity pulse irradiations. As a consequence, regarding dependency on the applied intra-line pulse distance, a change of the underlying coupling mechanisms can be supposed for the low-intense pulse regime, thus from inverse Bremsstrahlung absorption at a larger intra-line pulse distance to high-efficient optical energy transfer by plasma resonance absorption for the smaller ones. However, the underlying physical principles for the strong impact of the intra-line pulse distance causing up to 12-fold enhanced X-ray emissions even in case of low-intensity ultrashort pulses could not be clarified so far. Even though the interaction of the next following laser pulse(s) with the still apparent laser ablated nanoparticle plume at 1.6 MHz time scale could be a potential effect for the observed higher X-ray emissions. This was not considered yet, but evidence therefore can be found in the literature reporting on enhanced X-ray emissions from nanoparticles irradiated by ultrashort laser pulses [26,27].

### 3.5. Effect of the Temporal Pulse Distance on X-ray Emission in the Low-Intensity Pulse Regime

It becomes obvious that the temporal delay between the pulses will also affect X-ray emission when considering plasma resonance absorption as the dominant optical transfer mechanism for low-intensity pulses [28]. As already stated above, this is due to the fact that the actual shape of both the laser ablation plume and the critical plasma density layer is continuously changing with time. From this, it can be supposed that the impinging ultrashort pulses will meet different conditions in the interaction cross-section, depending on the inter-pulse delay defined by the PRF. Hence, the operating plasma density and actual flank angle are directly affected by the PRF, in turn, having an immediate impact on the optical energy transfer and further on X-ray photon yield.

**Figure 8:**
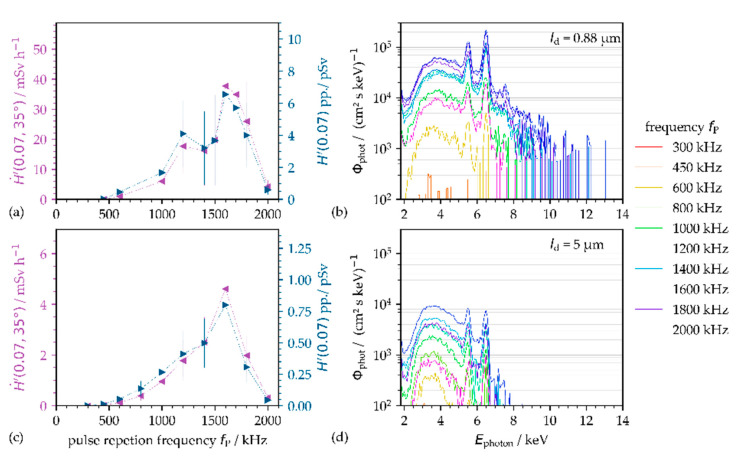
Effect of the pulse repetition frequency on X-ray emissions in the low-intensity pulse regime. Two intra-line pulse distances, (**a**,**b**) 0.88 µm and (**c**,**d**) 5.0 µm, were studied at 100 mm distance and 35° detection angle between the AISI 304 substrate and SILIX monitor. The dotted lines in (**a**,**c**) guide the eye.

With increasing PRF, which corresponds to shorter temporal inter-pulse delays, the X-ray photon emission increases to maximum level, as can be seen in Figure 8 for pulses of *I*_0_ = 1.6 × 10^13^ W·cm^−2^ irradiated at PRFs in the range of 1.6 MHz. A further increase of the applied PRF to 2 MHz caused a prompt drop of the X-ray photon yield. Here, too, a considerable impact of the intra-line pulse distance is recognizable when comparing the maximum X-ray dose rates presented in Figure 8a,c. At a 5.0 µm intra-line pulse distance, the maximum X-ray dose rate was measured of ${\dot{H}}^{\prime }\left(0.07\right)=4.6\;\mathrm{mSv}\cdot h^{-1}$, while 8-fold higher X-ray emissions of ${\dot{H}}^{\prime }\left(0.07\right)=37.7\;\mathrm{mSv}\cdot h^{-1}$ were detected at 0.88 µm, respectively. In addition, a significant difference of the X-ray emission characteristic can be seen in the corresponding X-ray photon spectra pointed out in Figure 8b,d. The X-ray photon spectra recorded at 5.0 µm intra-line pulse distance are characterized by broad continuous Bremsstrahlung emission in the photon energy range below 8 keV; see Figure 8b. At 0.88 µm in Figure 8d, by contrast, the X-ray photon spectra enlarged to 12 keV photon energy featured by dominant peaks of the characteristic X-ray emission lines. These results confirm the aforementioned change of the energy transfer mechanisms in the low-intense pulse regime from inverse Bremsstrahlung absorption to plasma resonance absorption. Thus, a strong influence of the actual conditions of the laser ablated plasma/nanoparticle plume on the effective laser beam absorption mechanism is suggested, which depends on both the temporal and spatial inter-pulse distance.

### 3.6. X-ray Emissions in the Burst and Burst-in-Burst Laser Pulse Regime

Laser pulse train processing in the burst and burst-in-burst (bi-burst) regime offers more flexibility to adjust and tailor optical energy deposition into the material. This is mainly due to the fact that the optical energy of a single irradiation event (burst) is divided by the number of individual pulses within the laser burst, while in the bi-burst regime, the individual pulses were further separated in sub-pulses. In the burst regime applied here, the time delay between the individual pulses within the burst was 15 ns and between the burst sub-pulses in the bi-burst regime was 440 ps, respectively. Two burst repetition frequencies were studied: PFR_B_ = 400 kHz and 1 MHz in bi-directional processing.

**Figure 9:**
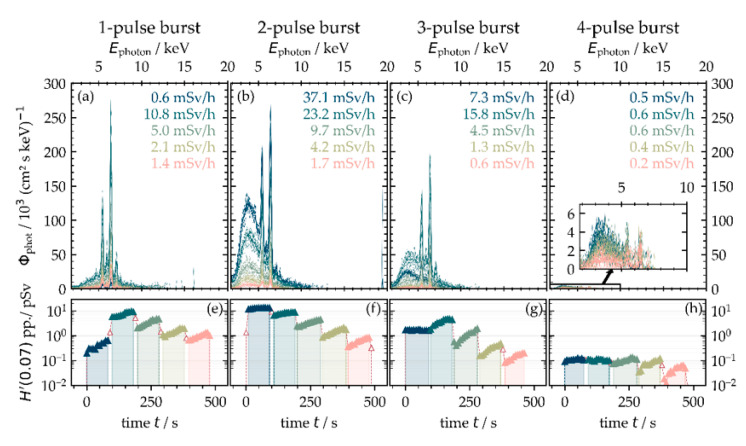
X-ray emission in the laser burst processing regime at a 400 kHz burst repetition frequency monitored at 100 mm distance, 35° detection angle, and five individual scans across the substrate. The intra-burst pulse number was varied between 1 (**a**) and 4 (**d**). The spectral X-ray photon flux and X-ray emission dose rate (**a**–**d**) as well as the X-ray dose per pulse (**e**–**h**) are presented for increasing number of scan crossings.

Initial studies on the burst mode in laser material processing showed a considerable drop of the material removal rate for each second irradiating pulse, especially when the next-following pulse was delayed to the previous one in the ten-nanosecond time domain [29]. Therefore, a reasonable explanation is the interaction of the second, fourth, sixth (and so on) intra-burst pulse with the ablation plasma/nanoparticle plume induced by the preceding pulse(s). Corresponding to the differences in material removal, a variance can also be observed for the X-ray emissions in the burst-mode regime monitored at a 35° detection angle and 100 mm distance between the substrate and SILIX detector; see Figure 9. For reference, the spectral X-ray photon flux and X-ray emission dose rate for 200 fs pulses of *I*_0_ = 3.8 × 10^13^ W·cm^−2^ applied in the single pulse (1-pulse burst) regime is presented in Figure 9a. At 400 kHz PRF, the full available power *P*_av_ = 33.0 W of the burst-mode laser was irradiated by repeated crossing (bi-directional, up to 5 scans) the substrate at 0.4 m·s^−1^ laser beam moving speed and a corresponding 1 µm intra-line pulse distance. The given X-ray emission spectra with a maximum of 12.5 keV photon energy are featured by distinct characteristic X-ray emission lines, and the Bremsstrahlung photon yield is relatively low. For the first scan crossing, the X-ray emission dose rate was measured to ${\dot{H}}^{\prime }\left(0.07\right)=0.6\pm 0.2\;\mathrm{mSv}\cdot h^{-1}$. This yields about 15 µSv·h^−1^·W^−1^ and is in the range of the dose rate levels presented above in Figure 8c for pulses at similar PRF (450 kHz) but longer pulse duration (600 fs instead of 200 fs). With ongoing processing, initially, the X-ray emission dose rate increased significantly in the second scan to ${\dot{H}}^{\prime }\left(0.07\right)=10.8\pm 2.0\;\mathrm{mSv}\cdot h^{-1}$ maximum. While in the further course of scanning, the X-ray emission reduced steadily to lower levels measured in the fifth scan of ${\dot{H}}^{\prime }\left(0.07\right)=1.4\pm 0.3\;\mathrm{mSv}\cdot h^{-1}$.

For two individual pulses in the laser burst (two-pulse burst in Figure 9b), a considerably higher amount of Bremsstrahlung emission was detected. This is potentially arising from the highly excited electron field induced by strong laser pulse with plasma interaction in the time domain of 15 ns, resulting in almost four-fold higher X-ray emissions than in the single pulse regime. It is noteworthy that such a high level of X-ray emission occurred with intra-burst pulses of half of the intensity of the single pulses (one-pulse burst, Figure 9a), because the impinging optical energy of the irradiation event is divided by the number of individual pulses in the two-pulse burst. By comparing these X-ray emissions with dose rates from pulses of similar intensity and PRF in Figure 8c, a major growth from 12.5 µSv·h^−1^·W^−1^ for single pulses to 1120 µSv·h^−1^·W^−1^ in the two-pulse burst regime can be recognized. These results confirm the strong plasma plume interaction in the laser burst regime with further evidence on plasma resonance absorption as the dominant energy transfer mechanism for pulses irradiating with very short time delay.

For a three-pulse burst, as shown in Figure 9c, the X-ray emission dose and Bremsstrahlung continuum originated somewhat in between single pulses and two-pulse burst irradiations. In this irradiation regime, the intensity of the individual intra-burst pulses reduced to one-third from the single pulses. With a higher number of pulses in a burst, the X-ray dose rates were found in the range of single pulses or below. This is exemplified in Figure 9d for laser bursts consisting of four intra-pulses. Accordingly, the intensity of the individual pulses reduced by a factor of four, which might be the reason for the Bremsstrahlung dominated X-ray emission spectra of low photon energy ranging between 2 and 7.5 keV.

For single-pulse processing at PRF = 1.0 MHz and P_av_ = 33.0 W average laser power, no X-ray emission could be detected, which might be due to the fact that the respective peak intensity *I*_0_ = 1.6 × 10^13^ W·cm^−2^ of the impinging pulses was too close to the threshold for X-ray photons generation on AISI 304. The observation is a bit in contrast to Figure 5, where the emission of X-ray photons can be seen for intensities even below of *I*_0_ = 1.0 × 10^13^ W·cm^−2^. However, this was achieved with pulses of 600 fs duration, while the pulse duration of the burst-mode laser presented here was 200 fs, which will also affect X-ray photon emission and equivalent dose rates.

**Figure 10:**
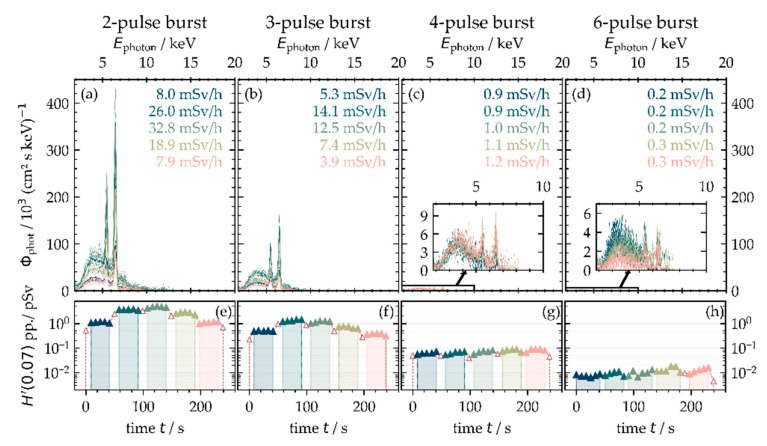
X-ray emission in the laser burst processing regime at 1.0 MHz burst repetition frequency monitored at 100 mm distance, 35° detection angle, and five individual scans across the substrate. The intra-burst pulse number was varied between 2 (**a**) and 6 (**d**). The spectral X-ray photon flux and X-ray emission dose rate (**a**–**d**) as well as the X-ray dose per pulse (**e**–**h**) are presented for an increasing number of scan crossings.

However, a significant enhancement of the X-ray emission dose rate accompanied by pronounced characteristic X-ray emissions were detected for laser bursts irradiating at a 1.0 MHz burst repetition frequency and corresponding lower peak intensity of the intra-burst pulses. The maximum X-ray emissions were measured to ${\dot{H}}^{\prime }\left(0.07\right)=32.8\pm 3.6\;\mathrm{mSv}\cdot h^{-1}\;$(3rd scan) with the two-pulse burst and ${\dot{H}}^{\prime }\left(0.07\right)=14.1\pm 1.6\;\mathrm{mSv}\cdot h^{-1}$ (2nd scan) with the three-pulse burst; see Figure 10a,b. AISI 304 processing with a higher number of intra-burst pulses yields much lower X-ray emissions, and the dominant Bremsstrahlung continuum shifted to X-ray photon energies below 7.5 keV, as can be seen in Figure 10d for the six-pulse bursts.

**Figure 11:**
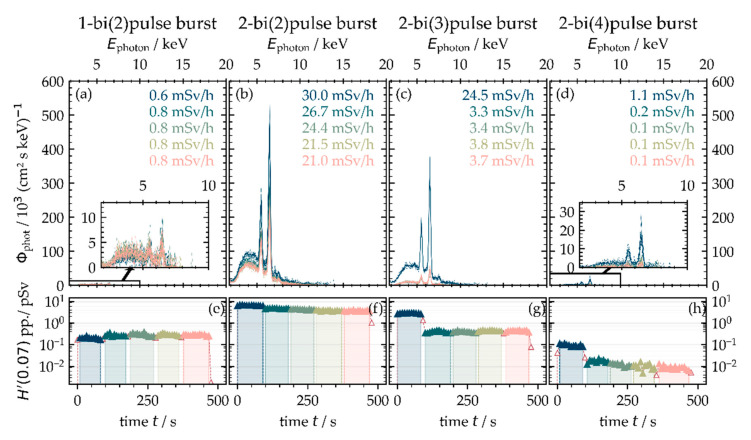
X-ray emissions in the laser bi-burst processing regime with notable X-ray dose rates at 400 kHz burst repetition frequency at 100 mm distance, 35° detection angle, and five individual scans across the substrate. The graphs display the high influence of the second MHz-burst pulse (two-pulse burst) with GHz bi-bursts (2–4 (**b**–**d**)) in contrast to the GHz bi-burst (**a**). The peak intensity of the sub-pulses was *I*_0_ = 1.9/0.95/0.63/0.48 × 10^13^ W·cm^−2^ The spectral X-ray photon flux and X-ray emission dose rate (**a**–**d**) as well as the X-ray dose per pulse (**e**–**h**) are presented for increasing number of scan crossings.

Another separation of the pulses in the bi-burst regime showed no further X-ray emission enhancement in the studied parameter range. For example, the X-ray emission dose rate was in the range between 0.6 mSv·h^−1^ < ${\dot{H}}^{\prime }\left(0.07\right)$ < 0.8 mSv·h^−1^ for a two-sub-pulse bi-burst of 440 ps inter-pulse delay at 400 kHz PRF_B_ and 33.0 W average laser power, as shown in Figure 11a. By irradiating two sub-pulses in a two-pulse burst processing scenario, such as shown in Figure 11b, the X-ray emission dose rate increased significantly to ${\dot{H}}^{\prime }\left(0.07\right)=30.0\pm 1.2\;\mathrm{mSv}\cdot h^{-1}$ maximum level, even when the peak intensity of the operating sub-pulses reduced to a quarter of the original single pulses below *I*_0_ = 1.0 × 10^13^ W·cm^−2^. However, with a higher number of pulses within the burst and bi-burst pulse train, the X-ray emission reduced to a lower value, which is mainly attributed to the corresponding lower peak intensity of the sub-pulses limited by the maximum energy of a laser burst; see Figure 11c,d.

However, these initial results obtained in the burst and bi-burst processing regime emphasize the great influence of laser pulse with plasma interaction for X-ray photon generation. In particular, the second intra-burst pulse is of great importance, causing strong enhancement of the X-ray emission dose rate. This will be proved in the ongoing study even for the case of ultrashort pulses in GHz-pulse trains at hundreds of Watts average laser power.

## 4. Summary and Main Conclusions

**Figure 12:**
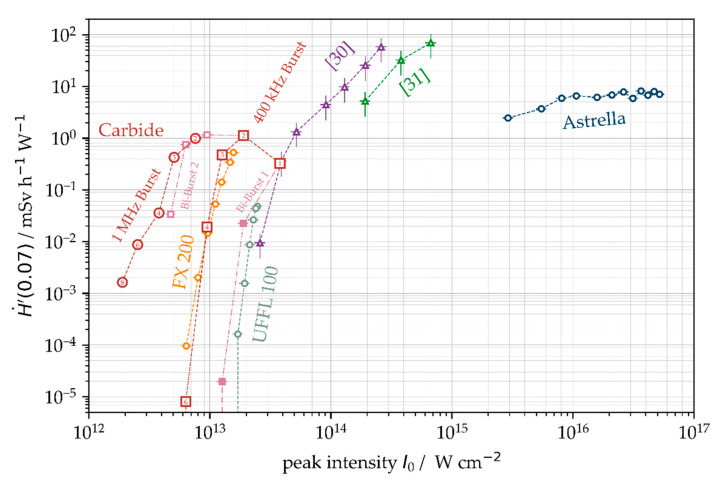
Summary of efficient X-ray emission dose rates as a function of peak intensity for different process regimes and laser systems. FX 200 (*f*_P_ = 1.6 MHz, *ld* = 0.88 µm); UFFL 100 (*f*_P_ = 506 kHz, *ld* = 0.88 µm); Astrella (*f*_P_ = 1 kHz, *ld* = 20 µm); [30] provided data recalculated for 100 mm detector distance, [31] provided data recalculated for 100 mm detector distance.

The presented study on laser-induced X-ray emissions on technical-grade AISI 304 stainless steel provides an overview on the laser processing under different conditions and parameter variations affecting the spectral X-ray photon flux and X-ray emission dose rate. In total, four different ultrashort pulse laser systems were applied providing complementary laser beam characteristics, including pulse peak intensities between 8 × 10^12^ W·cm^−2^ < *I*_0_ < 5.2 × 10^16^ W·cm^−2^, 2.0 MHz maximum PRF, up to 72.2 W average laser power as well as burst and bi-burst processing mode. The findings of this study confirm the general trend toward higher X-ray photon emission for pulses of higher peak intensity. Thereby, the monitored X-ray photon emissions are in good agreement with data reported previously by other groups; see Figure 12. The presented efficient X-ray emission dose rates were calculated from the X-ray emission dose rates monitored at 100 mm distance and 35° detection angle divided by the applied average laser power.

However, an unexpected high level of X-ray photon emission dominated by broad Bremsstrahlung emission could be detected for low-intensity pulses irradiation under specific processing conditions. For example, a maximum X-ray emission dose rate ${\dot{H}}^{\prime }\left(0.07\right)$ = 41.3 ± 2.8 mSv·h^−1^ and 0.4 mSv·h^−1^·W^−1^ corresponding to an effective X-ray emission dose were detected for pulses of *I*_0_ = 1.6 × 10^13^ W·cm^−2^ irradiated at 1.6 MHz PRF, 0.88 µm intra-line pulse distance, and 65 W average laser power. Therefore, a strong interaction of the next laser pulse(s) with the still apparent nanoparticle/plasma plume induced by preceding laser pulse ablations is suggested. As a consequence, plasma resonance absorption and effective electron plasma heating dominates the optical energy transfer, yielding broad Bremsstrahlung emission from the highly excited electron field. As a result, a considerable amount of X-ray photon emission could be detected even for low-intensity pulses at peak intensities considerably below the threshold value known so far. In summary, the following main conclusions can be stated on the basis of the presented results:

1. X-ray photon emission per pulse increases exponentially with peak intensity in the low-intense pulse regime (*I*_0_ < 1.6 × 10^13^ W·cm^−2^) and is on linear growth with high-intensity pulses (*I*_0_ > 10^16^ W·cm^−2^);
2. Unexpected high X-ray emission dose rates (${\dot{H}}^{\prime }\left(0.07\right)$ > 45 mSv·h^−1^) are achieved when low-intensity pulses irradiate at a small intra-line pulse distance and megahertz pulse repetition frequency (1.6 × 10^13^ W·cm^−2^, 1.6 MHz, 0.88 µm intra-line pulse distance);
3. Resonance plasma absorption and strong electron plasma heating are suggested as underlying process for highly excited electron fields yielding broad Bremsstrahlung spectra up to 12.5 keV X-ray photon energies and pronounced characteristic X-ray emission lines;
4. Within burst and bi-burst pulse trains, in particular, the second intra-burst pulses enhance significantly the X-ray emission dose rate that is potentially induced by strong laser pulse with plasma interaction.

With regard to kW-class ultrashort pulses lasers in materials processing, this unwanted X-ray emission can accumulate to harmful X-ray dose levels. In order to enhance the knowledge in this field, our actual research work is focused on high-average power lasers as well as megahertz PRF and burst-mode laser beams in order to understand and determine the dependencies of the characteristic laser beam properties on X-ray emission. Another future challenge is to develop and provide effective X-ray protection strategies, which is a mandatory prerequisite to pave the way for ultrashort pulse lasers as a powerful tool in modern micro fabrication.

## Figures and Tables

**Table 1:** Summary of the laser systems and studied laser parameter settings.

	Laser System	Symbol Unit	Astrella Coherent Inc.	FX 200 Edgewave GmbH	UFFL 100 AFS GmbH	Carbide Light Conversion
Parameter						
wavelength		*λ* in nm	800	1030	1030	1030
pulse duration		*τ*_H_ in fs	40	600	340	200
focus spot diameter		*d*_0,86_ in µm	13.5 **	30.0 ***	30.0 ***	49.7 **
pulse rep. frequency intra-burst frequency bi-burst frequency		*f*_P_ in MHz *f*_IB_ in MHz *f*_BB_ in GHz	0.001	0.2–2.0	0.51	0.4, 1.0 66.7 2.3
max. average power *		*P*_av_ in W	1.6	72.2	18.7	33
max. pulse energy *		*Q*_P_ in µJ	1600	36.1	37	82.5
max. peak power *		*P*_0_ in W	4 × 10^10^	5.6 × 10^7^	9.6 × 10^8^	3.6 × 10^8^
max. intensity *		*I*_0_ in W cm^-2^	5.2 × 10^16^	1.6 × 10^13^	2.7 × 10^13^	3.8 ×10^13^

* on target, ** LIU-plot method, *** MSM measurement.

**Table 2:** Chemical composition of the AISI 304 stainless steel alloying elements.

**Element**	C	Si	Mn	P	S	Cr	Ni	N
**Element Mass Fraction in %**	0.07	1.0	2.0	0.045	0.03	17.5–19.5	8.0–10.5	0.1

## Data Availability

Data sharing is not applicable to this article.

## References

[1] Roecker C., Loescher A., Delaigue M., Hoenninger C., Mottay E., Graf T., Abdou Ahmed M. (2019). Flexible Sub-1 ps Ultrafast Laser Exceeding 1 kW of Output Power for High-Throughput Surface Structuring. Advanced Solid State Lasers.

[2] Mueller M., Aleshire C., Klenke A., Haddad E., Légaré F., Tuennermann A., Limpert J. (2020). 10.4 kW coherently combined ultrafast fiber laser. Opt. Lett..

[3] Kamlage G., Bauer T., Ostendorf A., Chichkov B.N. (2003). Deep drilling of metals by femtosecond laser pulses. Appl. Phys. A.

[4] Gruner A., Schille J., Loeschner U. (2016). Experimental Study on Micro Hole Drilling Using Ultrashort Pulse Laser Radiation. Phys. Procedia.

[5] Mauersberger S., Schille J., Kujawa K., Schneider L., Million C., Hartung K., Oehlert K., Loeschner U. (2020). High-precision surface profiling using multi-hundred watts ultrashort pulse lasers and ultrafast polygon-mirror based scanner. J. Laser Micro Nanoeng..

[6] Gafner M., Kramer T., Remund S.M., Holtz R., Neuenschwander B. (2021). Ultrafast pulsed laser high precision micromachining of rotational symmetric parts. J. Laser Appl..

[7] Mueller F.A., Kunz C., Graef S. (2016). Bio-Inspired Functional Surfaces Based on Laser-Induced Periodic Surface Structures. Materials.

[8] Schille J., Schneider L., Mauersberger S., Szokup S., Hoehn S., Poetschke J., Reiss F., Leidich E., Loeschner U. (2020). High-Rate Laser Surface Texturing for Advanced Tribological Functionality. Lubricants.

[9] Legall H., Schwanke C., Pentzien S., Dittmar G., Bonse J., Krueger J. (2018). X-ray emission as a potential hazard during ultrashort pulse laser material processing. Appl. Phys. A.

[10] Legall H., Schwanke C., Bonse J., Krueger J. (2019). The influence of processing parameters on X-ray emission during ultra-short pulse laser machining. Appl. Phys. A.

[11] Behrens R., Pullner B., Reginatto M. (2019). X-ray emissions from materials processing lasers. Radiat. Prot. Dosim..

[12] Weber R., Giedl-Wagner R., Foerster D.J., Pauli A., Graf T., Balmer J.E. (2019). Expected X-ray dose rates resulting from industrial ultrafast laser applications. Appl. Phys. A.

[13] Freitag C., Giedl-Wagner R. (2020). X-ray Protection in an Industrial Production Environment. Photonics Views.

[14] Legall H., Bonse J., Krueger J. (2021). Review of X-ray exposure and safety issues arising from ultra-short pulse laser material processing. J. Radiol. Prot..

[15] Liu J.M. (1982). Simple technique for measurements of pulsed Gaussian-beam spot sizes. Opt. Lett..

[16] X-ray Mass Attenuation Coefficients, NIST STANDARD Reference Database 126.

[17] Gibbon P., Foerster E. (1996). Short-pulse laser—Plasma interactions. Plasma Phys. Control. Fusion.

[18] Schumacher D. (2012). Investigation of Laser Driven Hohlraum Radiation and Energy Loss of Heavy Ions in Indirectly Heated Plasma. Ph.D. Thesis.

[19] Sheng Z.M., Weng S.M., Yu L.L., Wang W.M., Cui Y.Q., Chen M., Zhang J. (2015). Absorption of ultrashort intense lasers in laser-solid interactions. Chin. Phys. B.

[20] Kraft S., Schille J., Mauersberger S., Schneider L., Loeschner U. (2020). Pump-probe imaging for process control and optimization in high-speed laser micro machining. Laser-Based Micro-and Nanoprocessing XIV.

[21] Cerchez M., Jung R., Osterholz J., Toncian T., Mulser W.P., Ruhl H. (2008). Absorption of Ultrashort Laser Pulses in Strongly Overdense Targets. Phys. Rev. Lett..

[22] Dragila R. (1983). Resonance absorption in inhomogeneous plasma with randomly rippled critical surface. Phys. Fluids.

[23] Catto P.J., Moore R.M. (1977). Sheath inverse bremsstrahlung in laser produced plasmas. Phys. Fluids.

[24] Chen L.M., Forget P., Fourmaux S., Kieffer J.C., Krol A., Chamberlain C.C., Hou B.X., Nees J., Mourou G. (2004). Study of hard X-ray emission from intense femtosecond:sapphire laser–solid target interactions. Phys. Plasma.

[25] Mulser P., Bauer D. (2010). Anharmonic resonance in intense laser-matter interaction: Key to collisionless absorption. AIP Conf. Proc..

[26] Mondal S., Chakraborty I., Ahmad S., Carvalho D., Singh P., Lad A.D., Narayanan V., Ayyub P., Ravindra G.K., Zheng J. (2011). Highly enhanced hard X-ray emission from oriented metal nanorod arrays excited by intense femtosecond laser pulses. Phys. Rev. B.

[27] Sankar P., Thomas J., Shashikala H.D., Philip R. (2019). Enhanced bremsstrahlung X-ray emission from Ag nanoparticles irradiated by ultrashort laser pulses. Opt. Mater..

[28] Horn A., Kaiser C., Ritschel R., Mans T., Russbüldt P., Hoffmann H.D., Poprawe R. (2007). Si-Kα radiation generated by the interaction of femtosecond laser radiation with silicon. J. Phys. Conf. Ser..

[29] Jaeggi B., Cangueiro L., Bruneel D., de Ramos Campos J.A., Hairaye C., Neuenschwander B. (2018). Micromachining using pulse bursts: Influence of the pulse duration and the number of pulses in the burst on the specific removal rate. Laser Applications in Microelectronic and Optoelectronic Manufacturing (LAMOM) XXIII.

[30] Legall H. (2020). Personal communication.

[31] Giedl-Wagner R. (2021). Personal communication.

